# SNAL: sensitive non-associative learning network configuration for the automatic driving strategy

**DOI:** 10.1038/s41598-022-24674-9

**Published:** 2022-11-21

**Authors:** Zhaoning Shi, Yong Zhai, Youtong Zhang, Hongqian Wei

**Affiliations:** 1 Low Emission Vehicle Research Laboratory, Beijing, 100081 China; 2 School of Mechanical Engineering, Beijing Institute of Technology, Beijing, 100081 China

**Keywords:** Habituation, Computer science

## Abstract

Nowadays, there is a huge gap between autonomous vehicles and mankind in terms of the decision response against some dangerous scenarios, which would has stressed the potential users out and even made them nervous. To efficiently identify the possible sensitivity scenarios, a new neural network configuration, named sensitive non-associative learning network (SNAL), is proposed. In such structure, the modulated interneurons, excited by abnormal scene stimulation for scene processing, are well processed and utilized to improve the training structure which refers to the sensitization mechanism in non-associative learning in neurobiology and the neural structure of Aplysia. When encountering the sensitivity scenes that the automatic driving agent is not good at or has not seen, the modulated interneuron facilitates the full connection layer neurons for the decision-making process, so as to change the final automatic driving strategy. In the process of constructing the model, a method to measure the similarity of the convolution feature map is proposed, which provides a new investigation tool for the properties of convolution networks after the feature extraction. Based on the Morris–Lecar equation in neurobiology, the dynamic model of modulating interneurons in the network is constructed. The automatic control optimization of the model is carried out by imitating the biological properties. The optimization method provides a reference for introducing neurobiological mechanism into deep learning and automatic control. To validate the effectiveness of the proposed method, the simulation test are executed and the existing methods are compared accordingly. The results show that the proposed SNAL algorithm can effectively recognize the sensitivity mechanism. Furthermore, compared with the existing algorithms, such as CNN, LSTM, ViT, the proposed algorithm can make better defensive strategies for potentially dangerous scenes rarely seen or not seen in the training stage. This sensitivity mechanism is more in line with the human driving intuition when dealing with abnormal driving scenes, and makes the decision more interpretable, significantly improving the traffic ability of autonomous vehicles under the sensitive scenes. In addition, this configuration can be easily combined with the existing mainstream neural network models and has good expansibility.

## Introduction

With the development of artificial intelligence, autonomous driving technology has attracted extensive attention since it benefits to human travel and reduce traffic accidents in theory. In the "DARPA challenge" in 2005, the team of Stanford University put forward the classic framework of "perception, planning, decision-making and control" for autonomous driving strategy^1^. Since then, most autonomous vehicles of Google and other companies have followed this framework and made improvements in technical details.

The decision-making part adopts the most reasonable automatic driving strategy according to the obtained scene information extracted by perception and the driving intention in planning process. For driverless vehicles, the decision-making should be able to respond in advance and avoid the possible risks according to the current, previous scene information or future driving intention.

A traditional decision-making method is the finite state machine^2^, which has been used in sequential circuits, communication protocols, and many other fields. Montemerlo et al.^3^ divided the driving states into 13 categories, and made decisions for a driverless system through the finite state machine. This method has good interpretability. This method constructs the mathematical model of state transformation function in different scenes and presents good interpretability; besides, it does not require complicated computational steps and is easily embedded into the real-time execution systems. However, this method utilized a rule-based decision process and is hard to deal with the sophisticated environments and possible abnormal phenomenon, which both limits its application in the real world. Therefore, to eliminate the flaws of the finite state machine on the fixed rules, some scholars investigate the Markov chain to deal with decision-making problems^4–7^. This method follows some statistical features and its decision comes from the probability of state transition, however, it can only observe the limited previous states, which would lead its poor decision strategy in some special cases.

**Figure 1:**
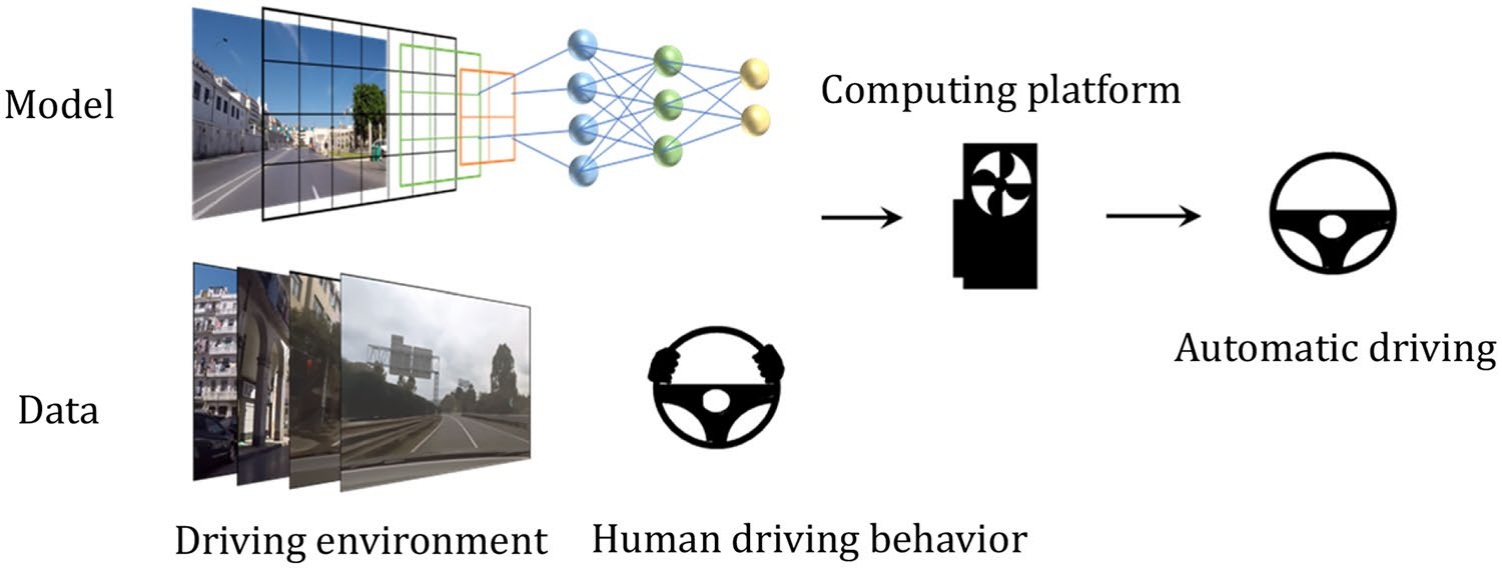
End-to-end autonomous driving learning mode. In the training process, the environmental information collected by the camera and other sensors is used as the input of the end-to-end agent of automatic driving model, and the driver's decisions (throttle, steering wheel angle, deceleration braking, etc.) are used as the training labels so that the end-to-end autonomous driving system can finally learn to output reasonable control decisions based on the surrounding scene information.

In recent years, with the rise of deep learning technology, the research on automatic driving decision-making using end-to-end learning methods has been developed^8,9^ as shown in Fig. 1. M bojarski et al.^10^ used CNN^11^ to directly map the camera input for the steering command; Morton et al.^12^ used Long Short-Term Memory (LSTM)^13^ for the control learning, which fully made use of the space–time information of the environment and obtained better performance than the baseline method; However, the causal logic of end-to-end learning from the input of environment information to the output for the control decision is hard to observe and explain. Inspired by the brain and LTC^14^, Mathias Lechner et al.^15^ designed a neural circuit policies (NCP) to obtain good performance, interpretability, and robustness with a very simple neural network structure.

An important factor restricting the autonomous vehicles is the driving safety. However, the sophistical environment and changeable scenarios really incur the identification difficulty for the abnormal situation. The control algorithm based on end-to-end learning can hardly complete the effective training for all real driving scenes due to the limitation of training data. Meanwhile, the end-to-end learning mainly makes decisions through the fitting principle rather than the effective logical reasoning, which may lead to the difference between automatic driving and human driving behaviors in some scenes. Due to the above shortcomings in the special environment or some extreme conditions, some scholars managed to improve the decision-making performance with advanced learning algorithms^16–19^, such as the reinforcement learning algorithm and game theory^20–25^. For human beings or other creatures, a considerable strength is the non-associative learning ability for the practical situation and the adaptiveness to the external stimuli. Non-associative learning is a basic concept of neurobiology. It mainly refers to the learning that does not form a clear connection between stimulus and response, or the neural activity process in which the individual's response to the stimulus gradually increases or decreases after a single stimulus act on the body repeatedly. It is divided into sensitivity and habituation. At present, there have been some studies on the habituation in neural networks^26^. However, as far as we know, this is the first time that the sensitivity mechanism in human being or other creatures is applied to the autonomous driving strategy. A weak noxious stimulus can cause a weak response, but if it occurs after a strong stimulus, the response of the nervous system to the weak stimulus may be significantly enhanced, which is called the sensitization effect^27^. There is no connection between strong stimulus and weak stimulus, nor does it require the combination of the two in time and space domain. This sensitization effect provides better protection for organisms to deal with external dangers. For instance, in the gill constriction reflex sensitization experiment of Aplysia, electric shock was given to the tail of Aplysia, and the subsequent response of Aplysia to siphon stimulation would be significantly enhanced. Although there is no logical connection between the two stimuli under natural conditions, after a strong stimulus occurs, the probability of the second stimulus occurring in a short time has showed an increasing trend. Therefore, this sensitivity is the result of biological long-term evolution (rather than the logical learning) to adapt to the danger of the external environment.

Inspired by the gill constriction reflex sensitization experiment of Aplysia, we developed the sensitive non-associative learning network configuration for the unmanned driving. Under the autonomous driving test the scenarios, where some dangerous situations are likely to have little or no data in the training stage because the probability of occurrence is not high, always exist in the real world since there is no perfect training process for autonomous vehicles, therefore, the end-to-end trained-well model may have poor ability to recognize the potential scenarios. To this end, we designed a neural network configuration with the function of scene abnormal feature recognition, which guides the autonomous decision-making module to recognize the unfamiliar abnormal scenarios, so as to adopt a more conservative driving strategy and improve the safety of driverless. Eventually, this function in the proposed SNAL changes the control strategy by extracting some information from the image information through the neural network responsible for perception. In the neurobiological principle of sensitive learning, this control is often realized by modulating interneurons^28^.

Generally, the proposed SNAL configuration in the autonomous driving strategy contributes to the following points.

1. This configuration can identify abnormal scenes that are rarely seen in the training stage or hard to handle for the trained agent, and accordingly affect the output of automatic driving decisions, which has avoided the potential accidents and improved the safety of automatic driving in the unknown environments.
2. This configuration can be easily extended to the existing neural network model for the autonomous driving decision-making process, which means that even if more advanced automatic driving models appear in the future, SNAL configuration can still be added to improve its decision-making performance for abnormal scenarios.

## Sensitive non-associative learning neural network model

**Figure 2:**
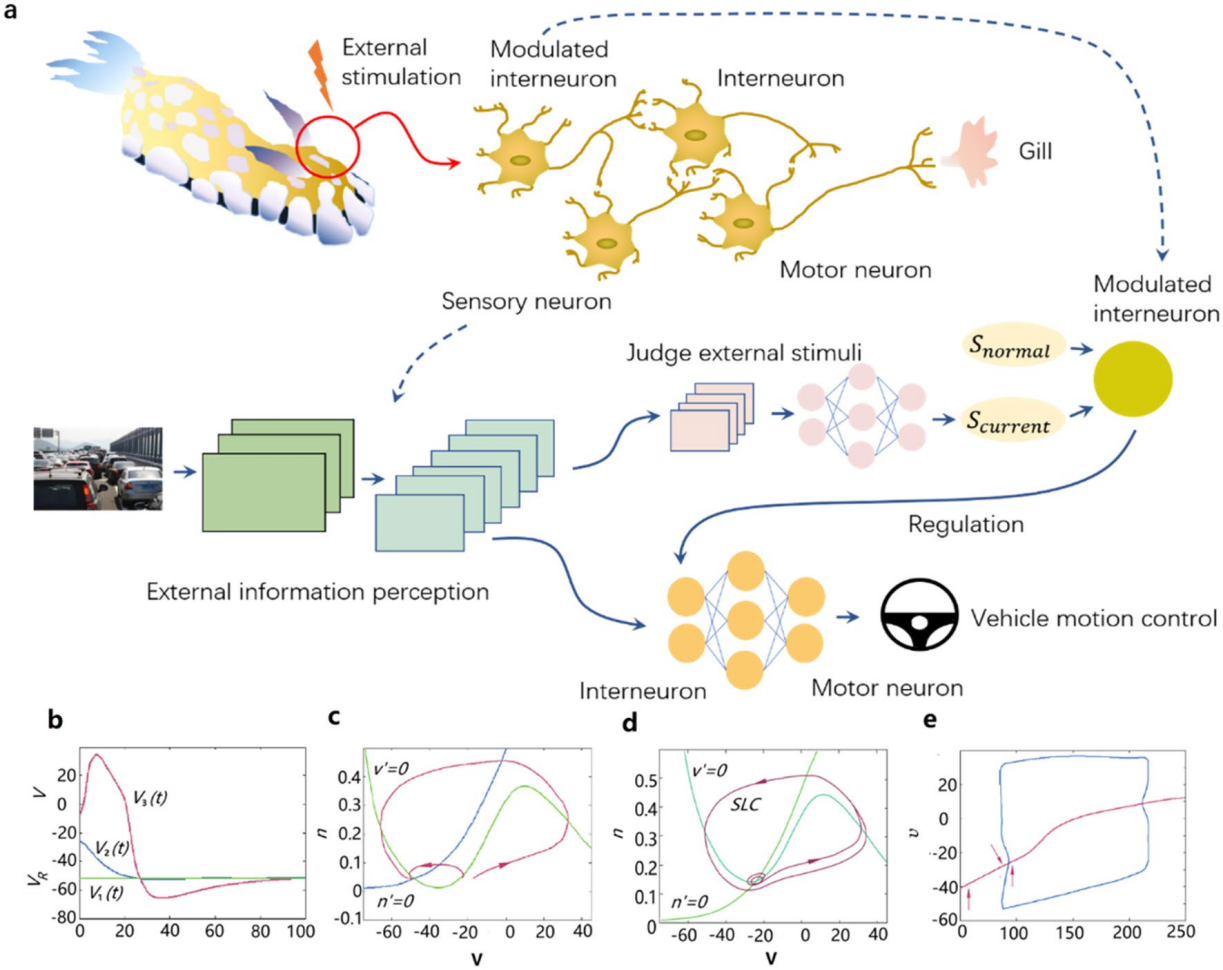
Sensitive non-associative learning neural network model. (**a**) Comparison of the nerve circuit of gill constriction reflex of Aplysia with the model in this paper. In the model, the convolution part is equivalent to the sensory neuron of the Aplysia; the modulation interneuron establishes the model alone, and the full connection layer of the lower half branch is equivalent to the interneuron, and the last layer is equivalent to the motor neuron. (**b**) Solution of Morris–Lecar equation, including the nerve potential curve when the external stimulus is insufficient to produce nerve excitation and the nerve potential curve when generating nerve excitation. (**c**) There is only one stable fixed point in the (V, n) phase plane. From the initial state, V and N will eventually return to the resting state. (**d**) There is a limit cycle in the phase plane. When the state converges to the limit cycle, it will oscillate and cannot return to the resting state. (**e**) Bifurcation diagram of ML model under Hopf bifurcation parameter. Voltage is a function of current. The curves above and below the fixed-point curve correspond to the maximum and minimum voltages along the periodic track respectively. The solid line represents the stable solution and the dotted line represents the unstable solution. For a more detailed explanation of figure b, c, d and e, please refer to^30^.

In order to enable the neural networks to produce a sensitive defensive behavior mechanism, an end-to-end autopilot control model is designed based on the neural structure of the sea rabbit. Its network configuration is shown in Fig. 2a. Compared with the existing depth-learning model, this model is devoted into responding to abnormal scenes that may hardly appear in the training stage. Explicitly, the network model extracts the features in the image and then establishes the relationship between the feature variables and driving strategies. Through the recognition of a strong feature (whether the feature alone has a great impact on decision-making process), the neural network would produce a stronger (generally more conservative) response to a common feature. For example, on the highway, it is a common decision to change lanes to overtake the car in front; however, when there is a big car driving nearby, the car changing lanes to overtake should become more conservative to prevent the danger from the big car, despite that the separate identification of the big car and the small car are two unrelated things.

An important problem to implement the system lies in the identification method of strong stimulus in the network model. Here, we define strong stimulus as an environmental variable which may be different from the training environment often encountered by the system itself. In order to make our system aware of this difference, in the upper right part of the network, a target recognition branch similar to yolov3^29^ structure is used to extract important features in the current road. In this way, compared with directly using the common convolution layer on the left to extract environmental features, its advantage is that the background of the environment often has great differences (such as lighting conditions, seasons, roadside buildings, etc.); however, these differences are not effective strong stimuli for the driving strategy. The effective strong stimuli could be abnormal cars, pedestrians, obstacles, etc. Therefore, through target recognition, we can ignore the background information in the image and turn the stimulated attention to the targets that are effective for the driving decision-making process. In addition, through target recognition, strong stimuli can also be refined. For example, large vehicles will be strong stimuli. The boundary box of target recognition can enable us to better quantify the changes in the size and quantity of the same kind of objects, so as to generate corresponding stimulus information.

The stimulus information designed in this paper is realized by identifying the current road object. For the road object target recognition, it is not required to have a high edge recognition accuracy but pays more attention to the overall intuitive information of quantity and volume. Therefore, this paper uses the bounding box parameters generated by the multi-target recognition to complete the construction of relevant information. Meanwhile, the absolute position information of the feature object should also be ignored. From a priori point of view, driving a car should focus on the difference between the number of objects (such as whether the road is crowded) and the size (such as the distance between big cars and small cars). This setting is also because these two indicators are easier to set and observe in the scenarios; therefore, the above features are utilized. However, more complicated features or environmental property could also be trained with our method. That is, to more concisely reflect the performance of the model, and more reasonable and accurate environmental indicators can be designed in actual projects. In this study or demo, to compare the above two differences between the current scenario and common scenarios, the following scenario similarity eigenvalues are defined:

1 $${\mathrm{S}}_{i}={\alpha }\cdot {\mathrm{N}}_{i}\cdot \overline{{n }_{i}}+\upbeta \cdot \sum \left({n}_{ij}-\overline{{n }_{i}}\right)$$

where ${\mathrm{S}}_{i}$ is the similarity eigenvalue of class *i* characteristic object, ${\mathrm{N}}_{i}$ is the number of recognition bounding boxes of class *i* objects. Here, this value is used to represent the number of objects in the current scene, and 0 is taken as the most common number, because it is obviously the safest and most familiar driving scene when there are no other vehicles; ${n}_{ij}$ is the bounding box area of the j-th individual of class i object; $\overline{{n }_{i}}$ is the average bounding box area of class *i* object in the previous training scene. The greater the difference between the above two parameters, the greater the value representing that the volume of the object in the scene image exceeds that of common objects. This setting makes it reasonable for large targets or multi targets to be far away from the driverless car, because a defensive driving strategy is not required for the long distance; For bounding box quantity items, multiply by $\overline{{n }_{i}}$ to make the order of magnitude the same as the latter one; α, β is the characteristic influence coefficient. The larger the α(≥ 0) is, the more sensitive the model will be to change in the number of objects in the scene. The larger the β(≥ 0), the more sensitive the model is to the supernormal volume of objects in the scene.

If the similarity eigenvalues S and $\overline{{n }_{i}}$ of common scenes are obtained by averaging ${\mathrm{S}}_{i}$ and ${n}_{ij}$ in the training set, the scene similarity index L of the whole current scene and common scenes can be defined as follows.

2 $$\mathrm{L}=\frac{1}{n}\sum {S}_{i}-S$$

The greater the similarity index is, the greater the difference between the current environment and the common environment is. This index acts as a stimulus signal in the modulated interneurons. The modulated interneurons are connected with the first layer of the full connection layer in the lower right of the figure through learnable weights, which can facilitate the construction of the neurons in this layer, so as to change the final control decision of the full connection neural network. The control method of modulated interneurons refers to the change law of neural state described by the Morris–Lecar equation^30^, as shown in Fig. 2b, which shows that small disturbance will quickly restore neurons to resting potential, and only large disturbance above a certain critical value will produce an action potential. We also hope that there is a critical value. The scene difference stimulation above this value will significantly increase the facilitation effect of modulated interneurons on the full connection layer, while below this threshold, the effect will be significantly reduced.

The original Morris–Lecar equation is:

3 $${C}_{M}\frac{dV}{dt}={I}_{app}-{g}_{L}\left(V-{E}_{L}\right)-{g}_{K} \cdot n \cdot \left(V-{E}_{K}\right)-{g}_{Ca}{m}_{\infty }\left(V\right)\left(V-{E}_{Ca}\right)\equiv {I}_{app}-{I}_{ion}\left(V,n\right),$$

4 $$\frac{dn}{dt}=\varnothing \cdot \left(\frac{{n}_{\infty }\left(V\right)-n}{{\tau }_{n}\left(V\right)}\right),$$

Among them,

$${m}_{\infty }\left(V\right)=\frac{1}{2}\left[1+\mathrm{tanh}\left(\frac{V-{V}_{1}}{{V}_{2}}\right)\right],$$

$${\tau }_{n}\left(V\right)=\frac{1}{\mathrm{cosh}\left(\frac{\mathrm{V}-{V}_{3}}{2{V}_{4}}\right)},$$

$${n}_{\infty }\left(V\right)=\frac{1}{2}\left[1+\mathrm{tanh}\left(\frac{V-{V}_{3}}{{V}_{4}}\right)\right],$$

where ${C}_{M}$ is membrane capacitance; ${I}_{app}$ is the applied stimulation current; ${g}_{L}$ is leakage conductance; ${g}_{K}$ is the conductance of K^+^ channel; ${g}_{Ca}$ is the conductance of Ca^+^ channel; ${E}_{L}$ is the leakage potential; ${E}_{K}$ is the Nernst potential of K^+^; ${E}_{Ca}$ is the Nernst potential of Ca^+^; $\varnothing$ control the dynamic speed of K^+^; V is the membrane potential; n is the gated variable, and its value is between 0 and 1; ${V}_{1}, {V}_{2}, {V}_{3}, {V}_{4}$ select the appropriate value to fit the voltage clamp data. Apart from V and n, the remaining parameters are constants. The Hopf term setting of M–L equation for specific values is listed in Table 1.

**Table 1:** M–L equation parameters.

Parameters	Hopf
$\varnothing$	0.04
${g}_{Ca}$	4.4
${V}_{3}$	2
${V}_{4}$	30
${E}_{Ca}$	120
${E}_{K}$	− 84
${E}_{L}$	− 60
${g}_{K}$	8
${g}_{L}$	2
${V}_{1}$	− 1.2
${V}_{2}$	18
${C}_{M}$	20

Equations (3) and (4) are written as follows.

5 $$\frac{dV}{dt}=f\left(V,n\right),$$

6 $$\frac{dn}{dt}=g\left(V,n\right),$$

For the above phase plane (V, n), it is required that there is only one stable fixed point (equilibrium point or resting point) without a stable limit cycle (which will cause periodic oscillation of action potential), so as to ensure that the intermediate neurons can finally return to the resting state after disturbance, as shown in Fig. 2c and d. According to Hopf bifurcation theory (as shown in Fig. 2e), when ${I}_{app}$ < 88.3 or ${I}_{app}$ > 217, there is only one stable fixed point in the phase plane^30^.

For the first layer of the decision-making full connection layer, they should not only deal with the characteristics of the flattened convolution layer but also accept the stimulation from the modulated interneurons to change their output. The calculation model is expressed as follows.

7 $$\mathrm{y}=\mathrm{g}(W1 \cdot X+w2\cdot V+b)$$

where $X$ is the input from the convolution layer; $\mathrm{g}$ is the activation function; $W1$ and $w2$ are obtained with the learning process, respectively.

According to the principle of gill constriction reflex of the Aplysia shown in Fig. 2a, the sensory neurons facilitated by the modulated interneurons are directly connected with the motor neurons through synapses; that is, the path from the neurons facilitated by the modulated interneurons to the final output is short, so that the effectiveness of facilitation can be guaranteed without using complex structures such as residuals. From the perspective of neural network training, the short back-propagation path is convenient to learn the appropriate $W1$ and $w2$ from a small amount of counterexample data. Therefore, referring to the effectiveness of Alexnet^31^ 3-layer full connection classifier, we select 3-layer for the number of full connection layers.

For external information perception, the common convolution layer is used. The features extracted by the common convolution layer should not only be used for multi-target recognition but also directly generate decisions through the full connection layer. Its weight uses the weight of the pre-trained YOLOv3 model.

## Experiment

### Model formulation and parameter setting

#### Data sets

**Figure 3:**
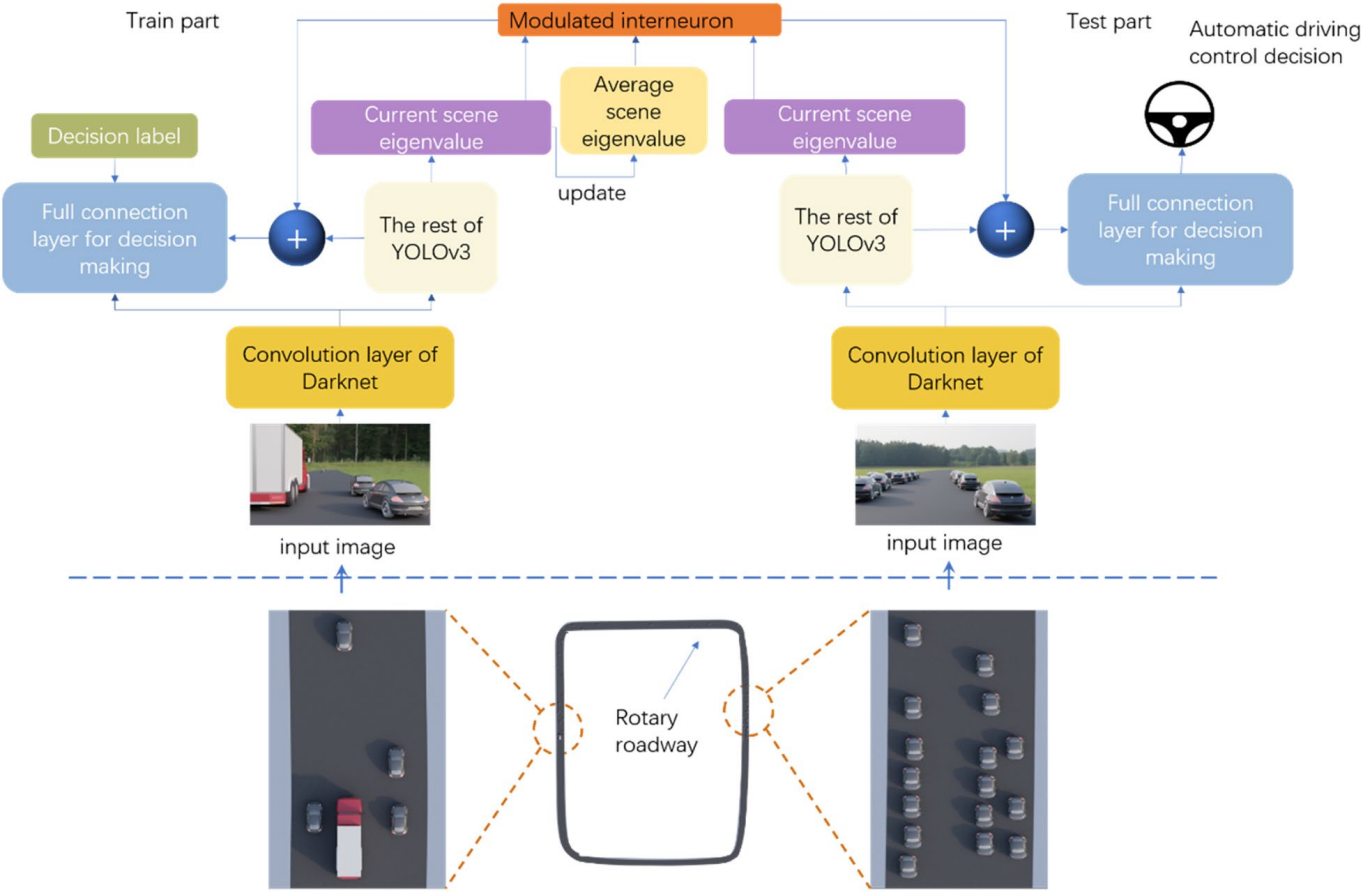
Schematic of the proposed SNAL. The upper part of the dotted line is the structure of the training and test model. The average scene eigenvalue in the figure can be updated gradually during the training process, or a pre-measured value can be directly specified. After the output of the modulated interneuron is spliced with the flattened Darknet output in the dimension, it is input to the decision-making full connection layer. The lower part of the dotted line is the simulated driving scene of the final performance test. There is an abnormal scene in the left and right sections of the circular road (the scene with trucks on the right and the scene with dense vehicles on the left), and the rest of the vehicles have normal density or locate on the open roads.

In order to set various working conditions conveniently and make the data sets close to the real driving scene, the circular road driving scene image produced and rendered by the Blender is used for the performance test, as shown in Fig. 3.

#### Model

As shown in the upper part of the dotted line in Fig. 3, all 53 convolution layers of darknet53 are used as the common convolution layer. The multi-target recognition part of scene feature extraction is the rest of Darknet's normal access to YOLOv3. The input dimension of the full connection part for decision-making is 1024 × 13 × 13 + 1 because the output of intermediate neurons occupies one dimension.

### Effectiveness analysis of YOLOv3 pre-training convolution head for CNN decision-making

In order to verify the modular and fast calling ability of SNAL to the existing neural network, and increase the training data of the feature extraction part, the whole pre-trained YOLOv3 network is used as the scene feature extraction part. Therefore, before directly using the Darknet pre-trained by YOLOv3 to connect the full connection layer for the decision-making task, it is necessary to prove that the Darknet part of YOLOv3 is still effective for the classification decision of subsequent MLP so that it is not required to consider the invalidity of convolution layer when adjusting subsequent parameters.

Since YOLOv3 and CNN perform different tasks, the convolution kernels of each layer after training must be different. However, both of them can classify objects, so they should have some similarity in the extraction of object features, such as extracting object edges. If the features extracted by the convolution kernel of both have sufficient similarity, the pre-training volume layer of YOLOv3 can support the subsequent access to the full connection layer for the decision-making process (similar to the classification task, so the feature map of CNN for classification is used for experiment below).

#### Calculation method of dissimilarity

As shown in Algorithm I, for the two feature maps, first match the overall mean value; that is, calculate the mean value of the pixel values of the two images respectively. If the mean value difference is too large (the maximum allowable mean value set in this paper is 0.5, and the pixel value is the normalized pixel value), it is directly considered that the two feature maps are completely different. Otherwise, add the difference between each pixel of the feature map with a small mean value to make the mean value of the two maps the same; Then, the down-sampling is used for noise reduction (in this paper, the pool of 3 × 3 is used for down-sampling, and the stride is set to 3); Finally, subtract the pixel values of the corresponding positions of the two feature maps, sum all the elements of the obtained difference matrix, and take the average of the map area to obtain the dissimilarity ds of the two maps. The smaller the value is, the higher the similarity of the two feature maps will be.
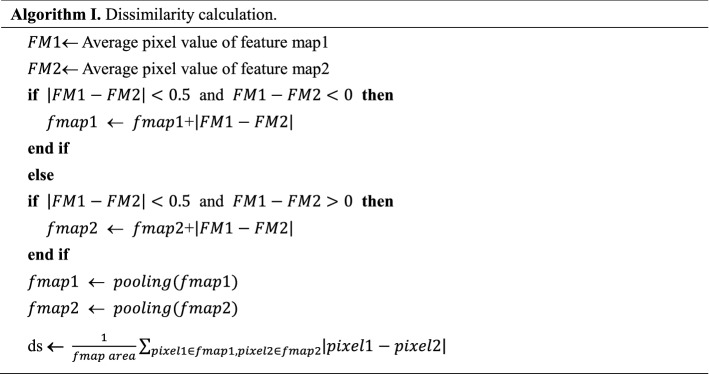


To analyze whether similar features have been extracted from the convolution layer of the same depth of the two networks, the dissimilarity of all feature maps of the same depth of the two networks is calculated. For each feature map in CNN, the feature map with the highest similarity in YOLOv3 (repeated correspondence is allowed) is found and the dissimilarity is calculated. If the dissimilarity is lower than the threshold M, it is considered that a similar feature has been extracted from the same layer of the two networks. Finally, the proportion of the image with similar features in all the feature images of the layer is calculated.

#### The setting of threshold M

**Figure 4:**
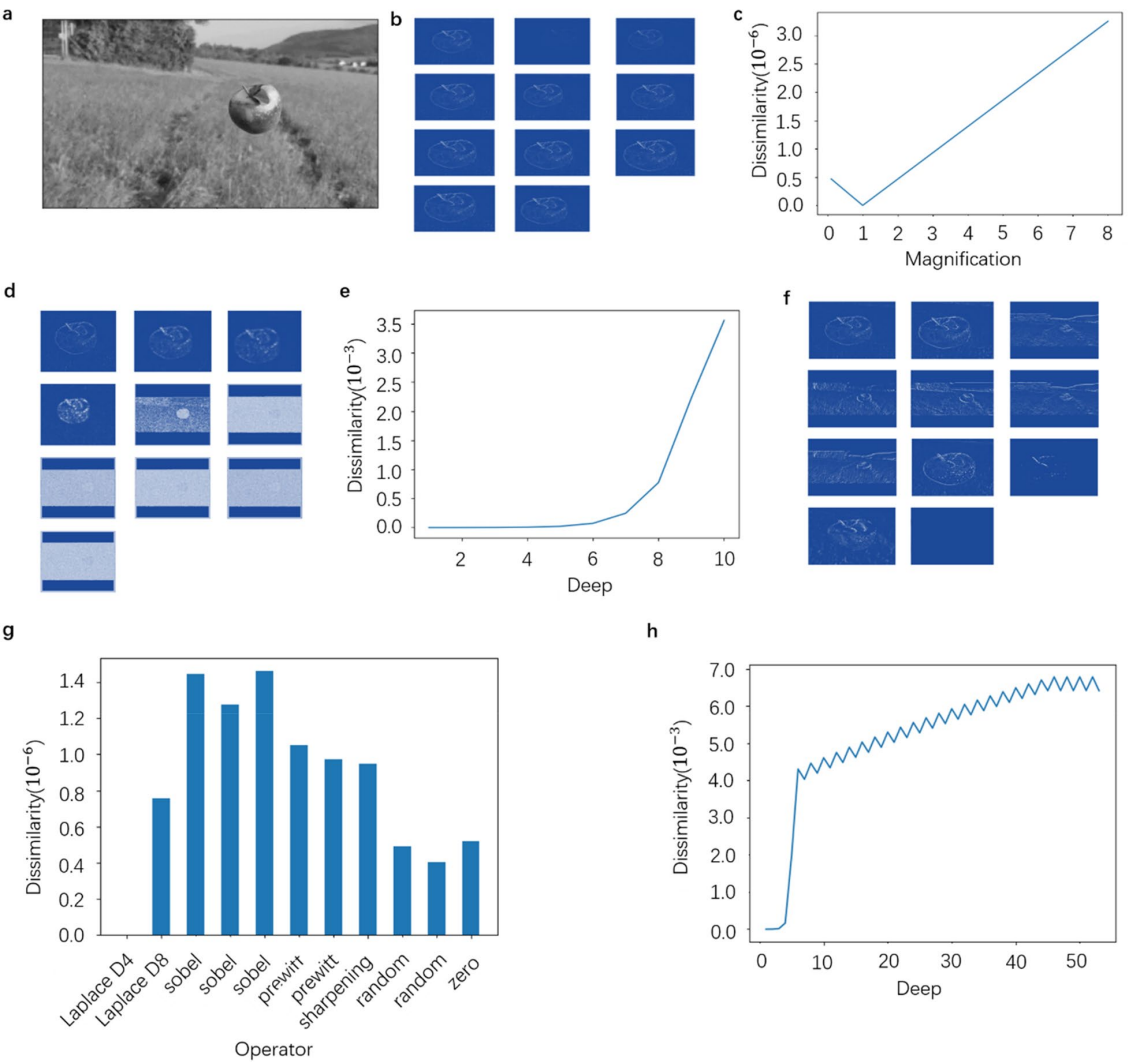
Similarity analysis of feature map. (**a**) Original image for feature extraction. (**b**) Feature maps of LaplaceD4 operator at different magnifications (from 0.1 to 8). (**c**) Variation curve of dissimilarity with different magnification. (**d**) Feature maps of LaplaceD4 operator at different depths (from 1 to 10). (**e**) Variation curve of dissimilarity with different depths. (**f**) Feature maps obtained by different convolution operators. (**g**) Dissimilarity of feature maps obtained by different operators relative to Laplace D4 operator. (**h**) At the end of the dissimilarity calculation, divide the depth value of the current feature map, and use 53 times Laplace D4 to simulate the 53-layers convolution network to obtain the curve of dissimilarity with depth. It can be seen that with the increase of depth, the final results after processing tend to converge.

In order to obtain a reasonable threshold M, a picture (as shown in Fig. 4a) is scaled and filled to the size of 416 × 416, and then input to Darknet and YOLOv3. Under the condition that both networks can correctly output the corresponding results (Darknet correctly classifies and YOLOv3 correctly identifies objects), use different convolutions to check its feature extraction, and output the feature maps for analysis. For comparison, we use a Laplace D4 operator as the benchmark convolution kernel, and the other convolution kernels will calculate the dissimilarity of the output feature maps. Figure 4b shows the feature maps obtained by the Laplace D4 operator under different magnifications (from 0.1 to 8) (local amplification is carried out for the unobtrusive features, the same as Fig. 4d and f). The change of the dissimilarity is shown in Fig. 4c. It can be found that the convolution kernel magnification and the dissimilarity basically form a fixed linear relationship. On the one hand, it shows that the calculation of the dissimilarity preserves the linear relationship of the feature map. On the other hand, it shows that we can accurately estimate the dissimilarity within a certain ratio range. For the feature maps extracted with the same convolution kernel for different times (i.e. different depths) as shown in Fig. 4d, the change of the dissimilarity is shown in Fig. 4e. It can be seen that the derivative of the dissimilarity to the depth increases, which shows that the dissimilarity can significantly distinguish the features extracted from different layers. At the same time, for the first six feature maps, the edge features of the apple are extracted, while the whole and background are extracted. The rising trend also reflects these differences. Finally, a variety of different operators are used as convolution kernels for comparison (the order is Laplace D4, Laplace D8, three Sobel operators in different directions, two Prewitt operators in different directions, two random filling operators with the mean value of 0, and the 0 matrix of 3 × 3). The obtained feature maps are shown in Fig. 4f, and the dissimilarity of different operators is shown in Fig. 4g. It can be seen that although the convolution kernels are different, the boundaries of apples are mainly extracted. Therefore, the dissimilarity is within a certain range. For the zero matrix, it can be regarded as the convolution kernel with a magnification of 0. From the above magnification relationship, it can be seen that the dissimilarity between it and the original convolution kernel is not large, which seems unreasonable. However, it can be considered that the value of the convolution kernel is very small, and the edge features of very small values are extracted; and the feature map is completely 0, which is only a special case with minimal possibility of occurrence.

By looking at the convolution kernels trained by the two networks, it can be seen that the numerical range of elements in both convolution kernels is 1 × 10^−4^ to 9.9 × 10^−2^. From the product of convolution kernel magnification 9.9 × 10^2^ and slope 0.5 × 10^−6^ in Fig. 4c, the dissimilarity of any two similar feature maps with depth 1 should not be greater than 5 × 10^−4^. Considering the great influence of depth on the dissimilarity, divide the depth value of the current feature map at the end of the dissimilarity calculation, use Laplace D4 53 times to simulate the 53-layers convolution network, and get that the dissimilarity changes with depth. As shown in Fig. 4h, it can be seen that the dissimilarity is limited to 0 to 0.007, and the change with depth after layer 6 is small, which shows that dividing by depth d is enough to limit the range of dissimilarity. Therefore, the dissimilarity of two similar feature maps with any depth d should not be greater than d × 5 × 10^−4^. Compared with depth and magnification, the influence of convolution kernel type, which can extract similar features on dissimilarity, would be ignored. Therefore, the threshold M is set to d × 5 × 10^−4^.

#### Validity analysis of convolution head

**Figure 5:**
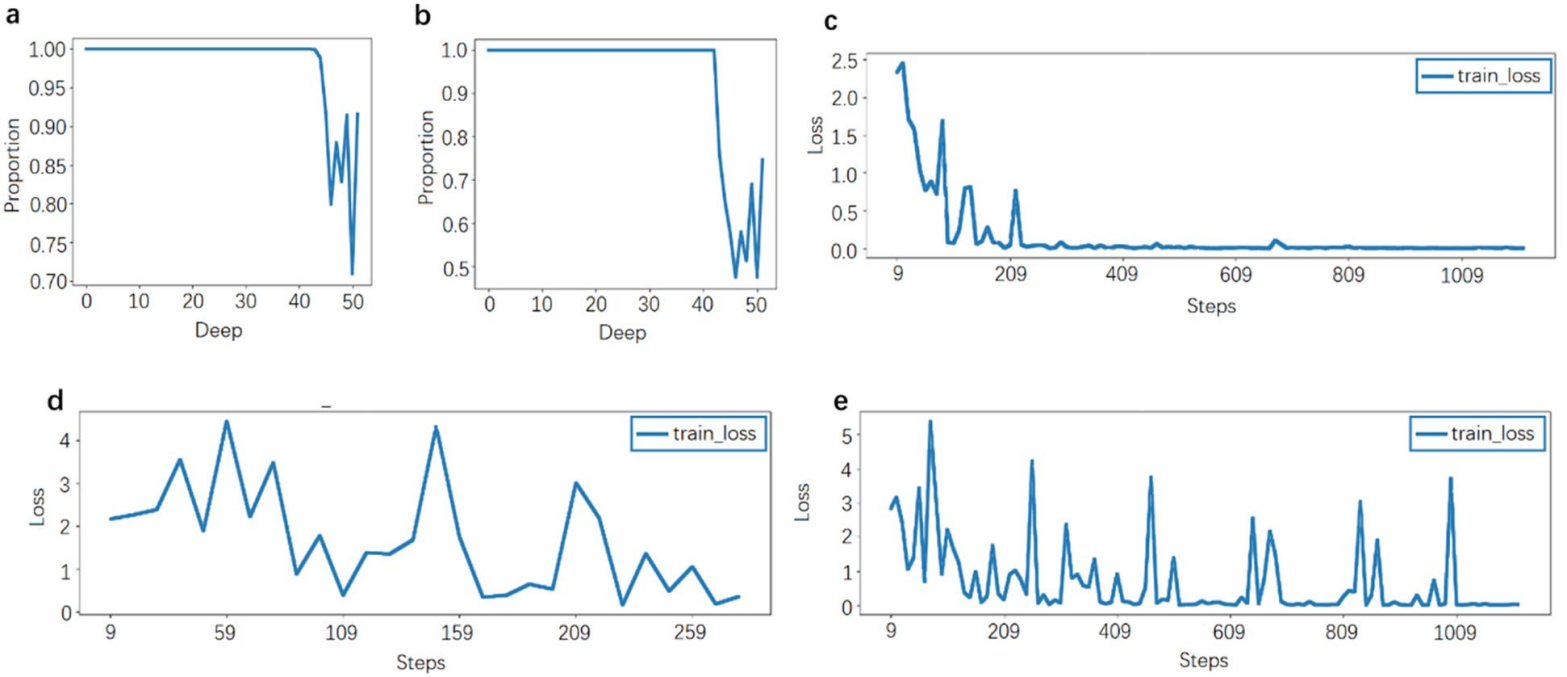
Effectiveness analysis results of convolution head. (**a**) Proportion of similar feature maps of each layer using threshold M. (**b**) The proportion of similar feature maps of each layer using threshold M/2. (**c**) The Darknet part of the pre-trained YOLOv3 model is used for the training convergence process of classification tasks. (**d**) The Darknet part of the untrained YOLOv3 model is used for the training convergence process of classification tasks. (**e**) Convergence of CNN training process using Darknet structure.

The above method is used to verify the similarity of each layer feature map of CNN and YOLOv3 constructed by Darknet53. Firstly, extract the output of each convolution part of the two network models Darknet. At the same depth, a CNN feature map is selected and compared with all the feature maps in YOLOv3. If the minimum dissimilarity is lower than the threshold M, it is considered that the CNN feature map has a corresponding similar feature map in the YOLOv3 model; that is, its corresponding features are extracted and displayed by the YOLOv3 model. As shown in Fig. 5a, when the threshold M is set as d × 5 × 10^−4^, each convolution layer of Darknet has a corresponding similar feature map, accounting for the proportion of all feature maps of this layer, and Fig. 5b adopts a more stringent threshold M/2. It can be seen that in both experiments, the proportion decreased significantly after 40 layers. The point of obvious decline is located in layer 43. The output size of this layer is 13 × 13, while the output size of the previous layer is 26 × 26, which is consistent with the intuition, because the feature map of 13 × 13 is directly related to the final decision-making and classification tasks. The feature map of the previous layer pays more attention to the feature extraction, and CNN and Yolo can classify objects in the image. Therefore, the extracted features should better have a great similarity. Even under the stricter threshold, about 50% of the feature maps are similar in YOLO. Therefore, it can be considered that using the Darknet part of pre-trained YOLOv3 to connect the full connection layer for decision-making is more effective.

#### Experimental verification

In the formal verification, the final convolution layer of Darknet in the yolov3 model pre-trained with coco data set is connected to the full connection neural network, which is effective for decision-making tasks. For the simplicity, a lightweight classification task of 10 objects (apple, bicycle, bowl, car, chair, cup, rabbit robot, spoon, table, truck) is utilized in this test. The number of 10 types of pictures is roughly same, with a total of 1450, of which 1070 are used as the training set and 380 as the test set, and the cross entropy is used as the loss function. The change of loss value in the training process is shown in Fig. 5c, which shows the changes of loss value after the Darknet convolution layer using the YOLOv3 pre-training parameters connected to the MLP. Figure 5d shows the change of loss value when the last convolution layer of the untrained YOLOv3 model is directly connected to the MLP for training without using the pre-training parameters, and Fig. 5e shows the change of loss value in the training process of CNN model using the Darknet structure. It can be seen that the MLP loss value of the pre-training model is small at the beginning stage, and then it can quickly converge to near 0, indicating that it is reasonable to use the pre-training YOLOv3 model to access the MLP. Without the pre-training stage, the model fluctuates cannot converge, indicating that MLP alone cannot complete the classification task when the convolution layer is unreasonable. The CNN model has a large loss value at the beginning and can eventually converge to a smaller value. On the one hand, it shows that the trained CNN is effective. In addition, it also reflects the advantages of the pre-training model, so that the model has a smaller loss value at the beginning step and can accelerate the convergence. The accuracy with the pre-training model, the untrained model, and the CNN model is 1.0, 0.071, and 0.795, respectively. It is reasonable that the MLP accuracy of the pre-training model is better than that of the overall training CNN because the model parameters pre-trained with the COCO data set can obviously provide more sufficient feature information than the training set with only 1070 pictures.

### Modulation interneuron establishment

**Figure 6:**
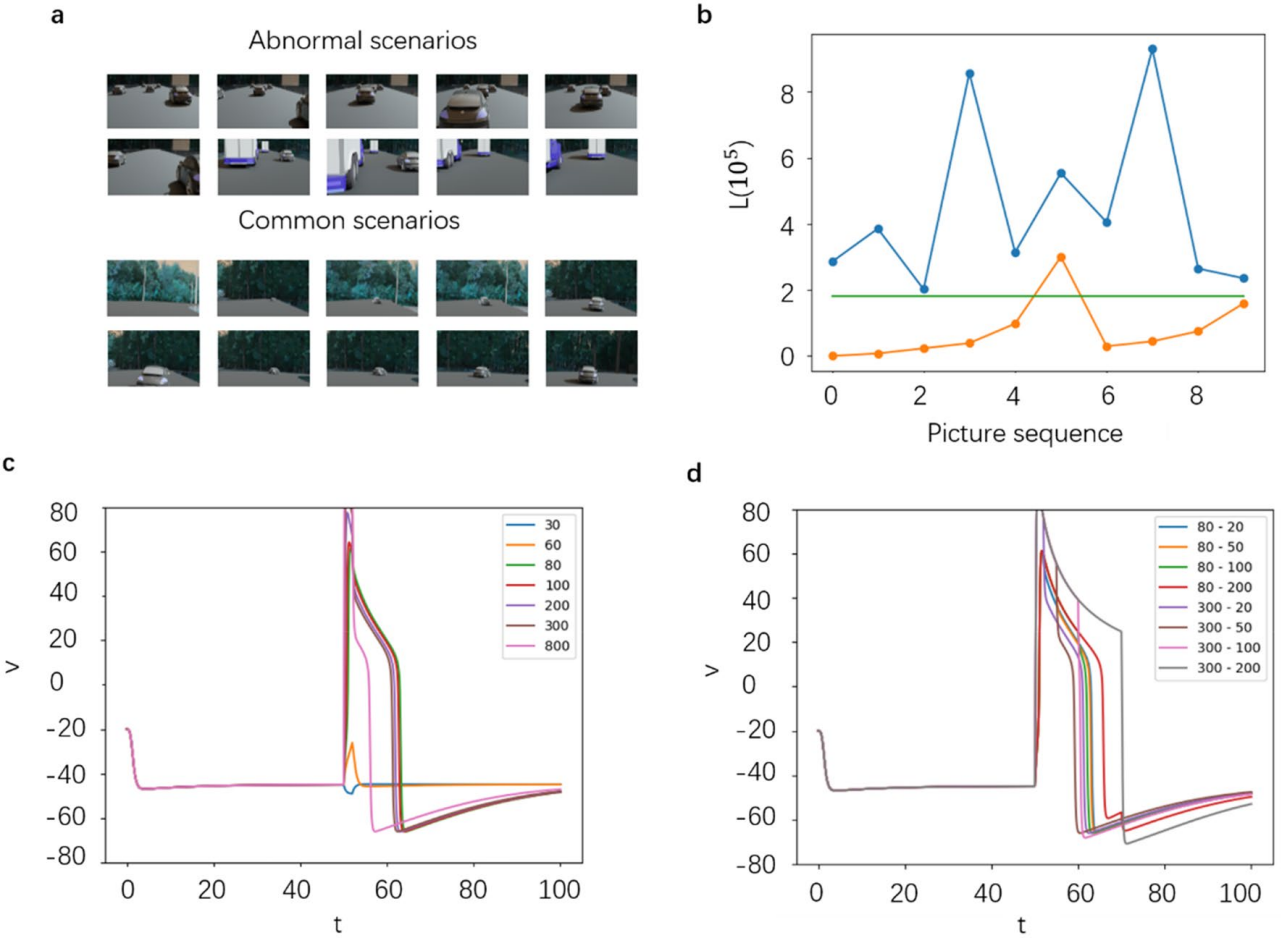
Modulated interneuron input and output. (**a**) Environment scene. (**b**) Scene similarity index under corresponding environment scene. (**c**) Excitation process of modulated interneurons under different values of applied current ${I}_{app}$. (**d**) Excitatory process of modulated interneurons under the different duration of applied current ${I}_{app}$.

The environmental characteristics output by YOLOv3 would be input into a modulating interneuron, which further outputs the regulation value to the first layer of decision-making MLP after processing. In order to obtain the eigenvalues of common scenes, we first use 150 images as common data, of which only a few images are unsafe driving environment scenes close to trucks. After calculating the environmental characteristic values of these scenes through Eq. (1), the mean value is taken as the common environmental characteristic value $S$, and the value of $\overline{{n }_{i}}$ in Eq. (1) is 47,332 (the boundary box parameter used is the value after projecting the output result of the neural network back to the original size of 1920 × 1080). Then, two abnormal working conditions (the first two lines of Fig. 6a) and 10 common working conditions (the last two lines of Fig. 6a) are used as the current environmental scenes. For simplicity, most of the common scenes here are those in which only one small car is at a medium and long distance. Then, the difference between the characteristic values of these scenes and the common environmental characteristic values $S$ is calculated through Eqns. (1) and (2) (that is, the scene similarity index L).

The similarity index L distribution between the scenes in the order of Fig. 6a and common scenes is shown in Fig. 6b. It can be found that taking the straight-line L = 180,000 in the figure as the boundary, two types of scenes can be clearly distinguished. However, it is noted that the number of vehicles corresponding to the third and last purple points (abnormal scenes) is relatively small in the abnormal scenes and the distance is far, so the L value is less than that of general abnormal scenes. In the image of the common scene corresponding to the sixth orange point, the small and medium-sized car is very close to the camera; therefore, the L value is greater than that of the general common scene.

In order to establish the dynamic model of modulated interneurons, the stimulation value and duration applied to them were studied. According to the binary M–L equation shown in Eqns. (3) and (4), the membrane capacitance parameter *C*_M_ is taken as 1 and ${I}_{app}$ is taken as 30 to 800 respectively, and when t = 50, it lasts for 2-unit times to obtain the change of potential with t as shown in Fig. 6c. When ${I}_{app}$ is small, the modulated interneuron potential returns to the resting potential immediately. For the applied current of 80 to 300, the neuron will maintain the excitation potential of 10 units of time and then return to the resting state. This stability is beneficial for the subsequent decision-making affecting MLP, because we can easily select the appropriate relationship between the step size of the difference equation and the frame rate of the experimental video. When the applied current is too high, the peak value of excitation potential is very large, but the duration will be very short. Therefore, when the environmental characteristic value L is used as the applied current, its excessive value should be limited. When the action time of ${I}_{app}$ is gradually extended to 20-unit times as shown in Fig. 6d, it can be seen that the excitation duration caused by smaller ${I}_{app}$ is almost stable. For a higher ${I}_{app}$, the excitation duration will be prolonged. In the experiment, in order to guard against abnormal scenes as much as possible, the threshold of common scenes is set as 100,000.

#### Modulation interneuron setting

**Figure 7:**
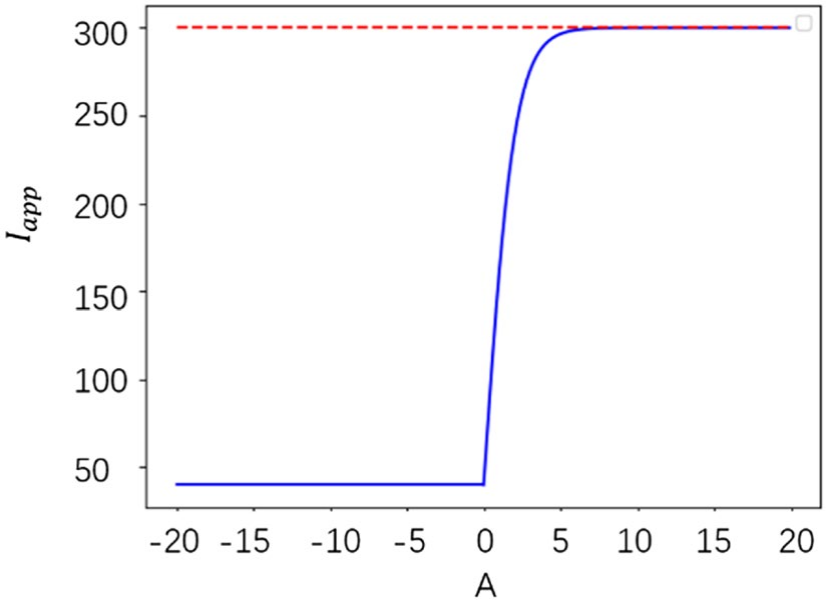
Ideal A and ${I}_{app}$ mapping diagram.

Finally, we hope that the modulated interneurons can output information in abnormal scenes to facilitate the decision-making of the first layer neurons of MLP, so as to make the decision conservative. Therefore, the abnormal value of the scene is firstly judged as follows.

8 $$A=L-100{,}000$$

when A is less than 0, it is considered that the current scene is a common scene that the existing neural networks could easily to handle. In the above equation, ${I}_{app}$ selects a smaller value of 40 so that the modulated interneuron does not generate excitation, so that its output to MLP does not change its decision results. When variable a is greater than zero, the current scene is considered as an abnormal scene. Thus, ${I}_{app}$ takes a larger value of more than 80. In order to limit the range of ${I}_{app}$, and simultaneously guarantee that when a is near 0, the response of intermediate neurons locates between abnormal and common, the ideal mapping relationship between a and ${I}_{app}$ is shown in Fig. 7.

The above figure can be obtained by translating and splicing a sigmoid function and a straight line. The formula is expressed as follows.

9 $${I}_{app}\left(A\right)=\left\{\begin{array}{c}\frac{520}{1+{e}^{-A}}-220, A\ge 0 \\ 40, A<0\end{array}\right.$$

The ${I}_{app}$ calculated above is input to the modulating interneuron, and the highest value of excitation potential in the four time steps after the stimulation is taken as the output of MLP. The number of this time step should be set according to the frame rate of time series image and the dynamic model of modulation interneuron excitation. In this paper, dt = 0.1.

#### Parameter setting

For this model, the input image resolution processing is 416 × 416, the learning rate is set to 0.0001, the Adam optimizer is used, and the cross entropy loss function ${L}_{CE}$ is used.

10 $${L}_{CE}=-\sum_{i}{P}_{i}\mathrm{log}{Q}_{i}$$

where ${P}_{i}$ is the actual probability of category i with a range of 0 or 1; ${Q}_{i}$ is its prediction probability ranging from 0 to 1.

### Analysis of comparative experimental results

**Figure 8:**
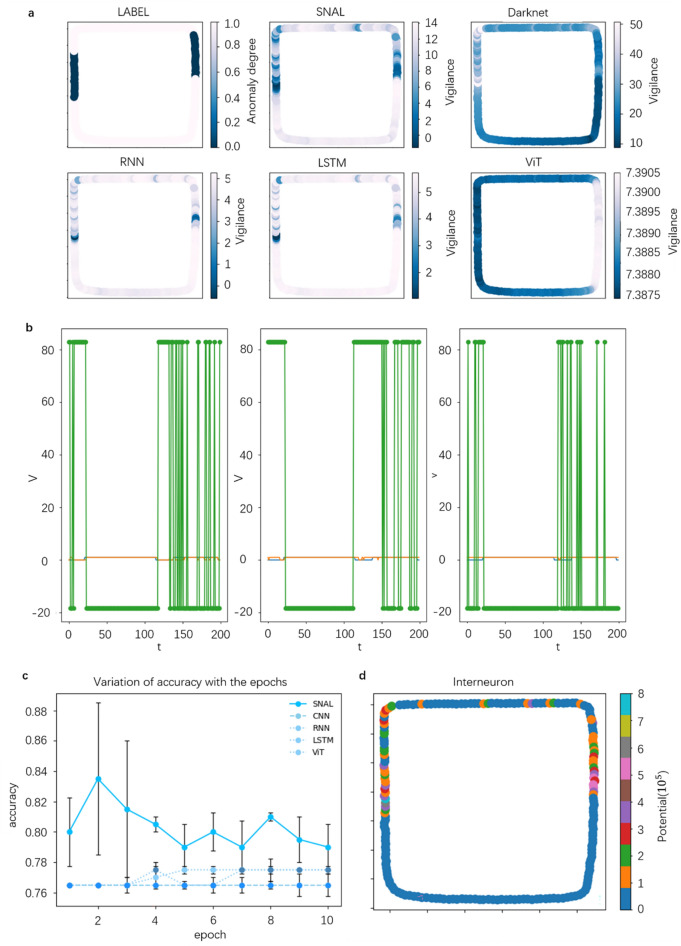
Experimental results and data. (**a**) Vigilance thermodynamic diagram of different network models. The darker the color, the more alert. (**b**) The excitation changes and classification results of modulated interneurons under the threshold of 100,000, 20,000, and 200,000 in common scenes, in which the green line is the excitation potential, the blue line is the label, and the orange line is the classification result (0 represents the abnormal scene and 1 represents the common scene). (**c**) Test accuracy of different network models and different training rounds. It can be seen that the effect of other comparison models has little difference, while the effect of the SNAL model is obviously better than that of the comparison model. (**d**) Thermodynamic diagram of scene eigenvalue change of SNAL model. It can be seen that the scene eigenvalue can distinguish different scenes well.

The proposed SNAL model is compared with other classical models. In order to better compare with YOLOv3 in SNAL, the CNN model uses the Darknet53 model, and the other models include RNN, LSTM, and ViT. The best results under different training and test sets are shown in Table 2, and the test results under different epoch training are shown in Fig. 8c. The first column in the table shows the accuracy of each model for the above 10 classification tasks. It can be seen that the accuracy of each model is higher than 0.9, indicating that all models are effective for blender modeling and rendering images. Moreover, SNAL's performance in classification is not inferior to other neural network models; The second column is the classification of safety scenes and abnormal scenes (scenes with a large number of carts and vehicles are considered dangerous abnormal scenes requiring conservative strategies, hereinafter referred to as scene classification), and its training set and test set are random scenes without sequence; The third column is the scene classification tasks with the sequential training set and test set; The fourth column is the scene classification tasks in which the training set without sequence and the test set with sequence; For the above training set scenes, there are 250 driving scenes, of which only 3 scenes have trucks, which are regarded as dangerous scenes, while the test set consists of 200 scenes, of which 47 are dangerous scenes (there are trucks and a large number of cars). This setting is to test the decision-making ability for abnormal scenes that do not often appear or even never appear in the training process. It can be seen that in all training and testing cases, the effect of the SNAL model is significantly better than that of other models, because 0.765 is actually the proportion of security scenes in the test set, so other models can hardly be vigilant against corresponding scenes when there are few abnormal data in the training set. When the training set is time-series data, the effect of SNAL is significantly worse than that of random data. Because there are too few abnormal data, their random appearance is more conducive to training than that of time-series data. See Table 3 for the comparison of model speed under the timing test.

**Table 2:** Comparison of accuracy of each model under different tasks.

Model	Classification	Safety decision	Safety decision	Safety decision
Darknet	0.976 (epochs = 3)	0.795 (epochs = 10)	0.765 (epochs = 10)	0.765 (epochs = 3–10)
RNN (32units)	0.984 (epochs = 7)	0.775 (epochs = 8–10)	0.775 (epochs = 7)	0.775 (epochs = 8–10)
LSTM (32units)	0.97 (epochs = 7)	0.765 (epochs = 3–7)	0.775 (epochs = 7)	0.765 (epochs = 3–7)
ViT	0.93 (epochs = 7)	0.78 (epochs = 7–10)	0.765 (epochs = 7–10)	0.775 (epochs = 8)
SNAL	1.0 (epochs = 3)	0.885 (epochs = 2)	0.795 (epochs = 2)	0.855 (epochs = 3)

1. non sequential scenario, 2. sequential scenario train and test, 3. sequential scenario test only.

**Table 3:** Comparison of test speed of each model.

Model	Darknet	RNN (32units)	LSTM (32units)	ViT	SNAL
Speed (fps)	14.8	12.33	12.36	16.99	10.89

In order to observe the effect of the model more clearly and intuitively, we will draw the vigilance heat map of the above scene classification in the simulation scene (as shown in Fig. 8a). The darker the color, the higher the vigilance of the model decision-making. SNAL can be vigilant against abnormal scenes rarely seen. RNN and LSTM can be vigilant in a few cases, but their effect is obviously inferior to the SNAL model. CNN and ViT cannot distinguish between common scenes and abnormal scenes. The poor effect of ViT may be related to too little data, which just reflects that SNAL can use a small amount of data to produce a good sensitivity mechanism. SNAL explicitly extracts the scene features through a neural network and generates expectations for its average value, and then compares the current scene features with the average expectations through a modulated interneuron as one of the bases for decision-making, which is a connection that is hard to learn by ordinary neural networks.

#### Influence of common scene threshold on model results

Figure 8B shows the excitation and final classification of modulated interneurons caused by setting the threshold of common scenes to 100,000, 20,000, and 200,000. When the threshold is set to 100,000, the excitation of modulated interneurons is basically synchronized with the change of actual labels, so that the decision-making MLP can well learn the correlation between the excitation of modulated interneurons and dangerous scenes, and then correctly identify abnormal driving scenes; When the threshold is set to 20,000, there are more scenes that can cause the excitation of modulated interneurons, which can improve the vigilance of automatic agents to the driving environment. However, the excitation mode and label in the training stage will have obvious asynchronous changes, which is hard for the decision-making MLP to learn the correlation between the excitation signal and the abnormal scene. Similarly, when the threshold is 200,000, it is hard to cause the excitation of modulated interneurons, and eventually lead to a radical driving strategy.

This also reflects the interpretability of the model. When a reasonable threshold is selected, the decision-making made by the whole model is highly related to the excitation of modulated interneurons, which shows that in the decision-making process, the inductive bias contained in Eqs. (1) and (2) is fully considered by the full connection layer, so that people can understand and control the influencing factors of model decision-making by adding different bias factors. More abnormal scene patterns, which cannot be fully trained, may activate modulated interneurons.

#### Classification potential of modulated interneurons

Equation (8) actually limits the more fine-grained scene perception ability of interneurons. Therefore, we recorded the changes of interneuron input in the scene without Eq. (8). For the thermodynamic diagram of loop working conditions as shown in Fig. 8d, the input of interneurons has more detailed changes rather than maintaining a few fixed values. If these properties can be reasonably interpreted and applied, a more detailed safety division can be made for the current driving environment.

## Conclusion

In order to solve the safety problem of abnormal scenes for the automatic driving decision-making, this paper proposes a sensitive non-associative learning network configuration. According to the structure and mechanism of the sensitive non associative learning nerve of the sea hare, we designed the structure of this configuration, and obtained the following conclusions through experiments.

1. This paper studies a new neural network configuration and realizes the sensitivity mechanism of neural network decision-making process in the autonomous vehicles. It is found that the new configuration with sensitivity mechanism makes much defensive decisions for dangerous scenes which is hard to realize with the existing CNN, RNN, LSTM, ViT, or other networks. This model can effectively improve the safety of driverless in abnormal environments. Meanwhile, it is also in line with the human driving thinking mode, improving the interpretability of driverless decision-making process.
2. The scene features in this paper only extract the number and volume features of vehicles in the scene, so it still cannot produce corresponding responses to abnormal scenes such as sunken roads. Therefore, the next focus of this model is to study a more reasonable description method of scene features and the judgment basis of sensitivity mechanism. Besides, the data processing method of inputting the output of one neural network as a regulation signal into the middle of another network is also a good exploration of the cooperation mode of multiple neural networks. SNAL configuration has a good combination; for instance, it can be easily embedded into other existing neural networks to produce various mechanisms and establish the connection between different things. For example, the rod-shaped features next to the road have a great connection with traffic signs and trees. By identifying the rod-shaped features and their location features and activating intermediate neurons, the recognition tendency of traffic lights and other objects can be improved. When there are no rod-shaped features, this recognition tendency decreases, which can more accurately distinguish whether the yellow circle in the sky is a yellow traffic light or the moon. And when combined with the most advanced network model, it hardly affects its accuracy.
3. In the process of network construction, we put forward the similarity judgment of feature maps and the method of imitating neuron potential for regulation, which provides a new reference for the research and establishment of neural networks. The application of SNAL in different tasks is worthy of further exploration.
4. Considering that the actual driving scenarios could produce more practical data and environment, the proposed SNAL configuration should be further validated in the real-world abnormal driving environments, and more detailed driving performance requires to be demonstrated in subsequent works.

## Data Availability

The main code generated and/or analyzed during the current study is available in the GitHub repository, https://github.com/TimeToLive404/SNAL-and-datasets.git. The datasets used and/or analyzed during the current study available from the corresponding author on reasonable request.
